# Histopathological Study of Changes in White-Pulp Structure Due to Lymphoid Depletion in the Spleen in Male Rats Caused by Exposure to Transitional Cigarette Smoke

**DOI:** 10.3390/toxics14020113

**Published:** 2026-01-26

**Authors:** Tyagita Hartady, Stevania Sifora, Ronny Lesmana, Brian Christian Sarniem

**Affiliations:** 1 Department of Biomedical Sciences, Faculty of Medicine, Padjadjaran University, Sumedang 45363, Indonesia; 2 Veterinary Medicine Study Program, Faculty of Medicine, Padjadjaran University, Sumedang 45363, Indonesia

**Keywords:** electronic cigarette, histopathology, spleen, tobacco cigarette, transition cigarettes

## Abstract

Conventional cigarette smoke and electronic cigarette vapor contain toxic compounds that may impair immune function, particularly in the spleen. This study evaluated histopathological changes in the spleen in male white rats (Rattus norvegicus, *n* = 32) divided into four groups: control, conventional-cigarette smoke (CCS), electronic cigarette vapor (ECS), and transitional cigarette smoke (TCS). The TCS group was sequentially exposed to CCS for 15 days followed by ECS for 15 days, with twice-daily exposure. Spleen tissues were analyzed semi-quantitatively using ImageJ and statistically using the Kruskal–Wallis test after Shapiro–Wilk normality testing. Comparisons among the four groups showed significant differences in necrosis (*p* = 0.025) and vascular degeneration (*p* = 0.027). In contrast, hemosiderin, congestion, stretching, and vacuolization parameters did not show statistically significant differences among groups (*p* > 0.05). These findings suggest that switching from conventional cigarettes to e-cigarettes does not protect against splenic damage and may exacerbate immune dysfunction due to cumulative toxic exposure.

## 1. Introduction

Animal and environmental health must be considered in maintaining balance by avoiding exposure to harmful biological, chemical, and toxic agents [1]. An example of an environmental toxin is cigarette smoke produced by smoking. Smoking has become widespread globally and nationally [2]. According to the Global Adult Tobacco Survey (GATS) conducted by the Indonesian Ministry of Health, the prevalence of smokers reached 33.8% (i.e., 1 in 3 Indonesians are smokers), and it was especially high in terms of electronic smokers, increasing from 0.3% (2011) to 3% (2021). Meanwhile, the overall number of adult smokers in this 10-year period was 60.3 million in 2011, with up to 69.1 million smokers in 2021. Based on the results of the 2023 Indonesian Health Survey (SKI), the estimated number of active smokers has reached 70 million people, with smokers aged 10–18 years amounting to around 7.4% [3]. Tobacco cigarettes and electronic cigarettes contain hazardous substances that potentially cause health problems. Tobacco cigarettes are widely consumed and contain nicotine, tar, carbon monoxide, cadmium, benzene, ammonia, formaldehyde, acetaldehyde, and other toxic compounds [4], while electronic cigarettes contain aldehydes, tobacco alkaloids, propylene glycol (PG), polycyclic aromatic hydrocarbons (PAHs), Tobacco-Specific Nitrosamines (TSNAs), Phenolic Compounds, Volatile Organic Compounds (VOCs), and others [5]. Smoking not only affects active smokers but also individuals around them, including animals that become passive smokers. This study used male rats (*Rattus norvegicus*) to evaluate the effects of cigarette smoke exposure on the immune system, especially the spleen, which plays a role in the body’s immune response. The spleen is a large peripheral organ in the immune system and acts as an immunological filter [6]. Histologically, the spleen consists of two parts: stroma and parenchyma [7]. The stroma has two parts: a capsule and trabeculae. The parenchyma is divided into two parts: the red pulp, which is composed of macrophages, plasma cells, and blood elements, functioning as a filter for old or damaged red blood cells, and the white pulp, which consists of densely arranged lymphocytes surrounding a central artery, playing a key role in immune defense [8]. Cigarette smoke contains substances that form free radicals, which activate inflammatory cells such as neutrophils and macrophages. These produce reactive oxygen species (ROS), leading to tissue damage [9]. A foreign substance will be detected as an antigen to be phagocytosed, leading to the secretion of cytokines, followed by the attraction of neutrophils to the site of inflammation. Systemic inflammation can overburden the spleen, leading to swelling and tissue damage, which may impair immune responses [10]. Cigarette smoke also directly induces inflammation and oxidative stress, reducing immune efficiency and contributing to immune system disorders. A study reported that e-cigarettes are considered safer because they do not contain nicotine, and this is an influencing factor for many individuals who switch to e-cigarettes [11,12]. Research on the impact of cigarette smoke on the spleen is still very rare.

This study aims to observe changes and differences in spleen histopathology in rats exposed to tobacco cigarette smoke, electronic cigarettes, and a transition from tobacco cigarettes to electronic cigarettes. *Rattus norvegicus* was chosen as the experimental model because rodents are widely used due to their genetic similarity to humans and suitability for toxicological studies [13].

## 2. Materials and Method

### 2.1. Ethical Considerations

This study was approved by the Ethics Commission of the Faculty of Medicine, Padjadjaran University (No. 915/UN6.KEP/EC/2024), on 19 August 2024.

### 2.2. Study Design

The sample was divided into four groups (*n* = 8 per group): a control (K), with no smoke exposure; a conventional-cigarette smoke (CCS) group; an electronic cigarette smoke (ECS) group; and a transitional cigarette smoke (TCS) group. The CCS group was exposed to conventional tobacco cigarette smoke generated from commercially available unfiltered clove cigarettes (“DC-K”). Each cigarette contained 2.4 mg of nicotine and 38 mg of tar, with dark-brown tobacco and 12 cigarettes per pack. Exposure was standardized to three cigarettes per session, with an estimated exposure duration of approximately 10 min per session, conducted twice daily (morning and afternoon) for 30 days.

The ECS group was exposed to electronic-cigarette vapor generated using a commercially available electronic cigarette device (“GPK” V1; FM). The device was operated at a power range of 15–25 W, utilized a tank-based vapor system with cotton and coil (0.5 Ω resistance), and had a maximum liquid capacity of 2 mL, with a battery capacity of 1300 mAh. The e-liquid used was a grape-flavored formulation (“Fr-Lo”), obtained from VO (Jakarta, Indonesia), containing 50% propylene glycol (PG), 50% vegetable glycerin (VG), 0 mg of nicotine, and natural and artificial flavoring agents. In each ECS exposure session, 0.6 mL of e-liquid was used, with an estimated exposure duration of approximately 10 min per session, conducted twice daily for 30 days.

The TCS group was exposed to conventional-cigarette smoke for the first 15 days following the CCS protocol, followed immediately by electronic-cigarette vapor exposure for the subsequent 15 days following the ECS protocol, without any recovery or washout period between the two exposure phases.

For all exposure groups, cigarette smoke or vapor was delivered into the exposure chamber using an air vacuum system. After each exposure session, the vacuum system was turned off, and artificial ventilation was applied to remove residual smoke or vapor. The rats were then removed, and the exposure chamber and vacuum apparatus were cleaned to eliminate residual contaminants prior to the next exposure session.

Rats were randomly selected and assigned to their respective experimental groups for 30 days. Each group consisted of eight rats, with a total of 32 rats. Each rat was marked with a permanent marker with a different color according to its group. The experimental unit in this study was a rat, which was placed in a cage with a minimum size of 1.497 cm^2^ that was designed for eight rats. The cage was well ventilated and equipped with a 12 V, 12 cm fan for air circulation when needed. Rats were given pellet food and as much water as they wanted.

According to the inclusion criteria, we included healthy male *Rattus norvegicus* specimens (as determined before the study), rats that were able to complete the entire exposure procedure, and rats that did not have disabilities. Exclusion criteria included a weight of less than 200 g or more than 300 g. Final body weights ranged from 230 to 350 g, with a mean of 267.91 g. Histogram and box plot analyses demonstrated no significant differences in body weight among the experimental groups (*p* > 0.05), indicating that body weight did not confound the evaluation of splenic histopathological outcomes. The selected weight range ensured physiological consistency and experimental reliability. Inclusion criteria comprised being a healthy male *Rattus norvegicus* that successfully completed the exposure protocol, whereas animals with body weights <200 g or >300 g at baseline were excluded. All animals were housed under controlled laboratory conditions with a regulated temperature and routine sanitation to maintain hygienic conditions and minimize the risk of disease. The rats were placed in the Diagnostic Laboratory, Veterinary Teaching Hospital, Padjadjaran University. The laboratory temperature was gradually adjusted from 28 °C to the target temperature of 25 °C with the assistance of wood husks. The food container (Citrafeed^®^ RatBio Feed, Jakarta, Indonesia) and water dispenser were cleaned every morning and evening with running water and dried to ensure cleanliness and minimize the risk of disease transmission via feces and urine contamination.

In this study, we used a true experimental design with controlled interventions. The experimental groups included a control group that was only exposed to clean air, a group exposed to tobacco smoke, a group exposed to nicotine-free e-cigarette vapor (50% VG and 50% PG), and a transition group that was initially exposed to tobacco smoke and then switched to e-cigarette vapor. The test animals were placed in cages designed according to the recommendations of the University of North Carolina at Chapel Hill (2016) [14], equipped with a ventilation system with an air flow rate of 2 Lpm and an artificial fan for air circulation.

Exposure to tobacco smoke was conducted using a modified inhalation device adapted from Hage et al. (2017) [15], equipped with a vacuum pump (RS555) generating a suction flow rate of 15 L/min. The cigarettes used in this study were clove cigarettes with the trademark initials “DC-K,” containing 2.4 mg of nicotine and 38 mg of tar per cigarette. A total of six cigarettes were combusted per day, and the resulting smoke was delivered to the exposure chamber via a flexible tubing system. The selection of this type of cigarette and exposure was based on reports indicating that the average daily cigarette consumption among the Indonesian population is approximately 8–9 cigarettes [16]. Commercially available cigarettes typically contain 1.1–1.8 mg of nicotine per cigarette, corresponding to an estimated daily nicotine intake of 8.8–16.2 mg. Therefore, the use of DC-K cigarettes in this study was considered appropriate to approximate typical nicotine exposure levels in active smokers.

For e-cigarette exposure, the model “GPK” was used, which utilizes a tank system to produce vapor from 50% VG and 50% PG liquid without nicotine. The controlled vacuum system ensured exposure at a flow rate of 4 Lpm. The smoke inhalation system consisted of a vacuum pump, a digital automatic timer, a flow meter used to monitor air flow, and a piping system with stainless-steel valves for controlled air distribution. After the exposure period ended, the rats were euthanized in accordance with ethical guidelines for animal research. The spleen was analyzed histopathologically in the veterinary laboratory. The analysis included tissue processing, staining, microscopic evaluation of pathological changes, and comparison between groups. All procedures were carried out in accordance with ethical research standards at Padjadjaran University to minimize stress on test animals. Termination went smoothly, and no undesirable incidents, such as termination failure or errors in performing the anesthetic agent injection procedure or cervical dislocation, occurred.

### 2.3. Euthanasia Techniques After Exposure to Cigarette Smoke

Euthanasia was performed using Ketamine and Xylazine preparations at doses of 30 mg/kg and 3 mg/kg. Ketamine and Xylazine were used as anesthetics to eliminate pain that may arise during termination. Then, euthanasia was carried out via cervical dislocation.

### 2.4. Spleen Histopathology Group

In the histopathology study, we used toxin-containing biological material derived from the spleens of 32 male rats (*Rattus norvegicus*) aged ten weeks and weighing 200 g that had been exposed to tobacco smoke, e-cigarettes, and a transition from cigarette smoke to e-cigarettes. The material was stained using hematoxylin and eosin (H&E) to enable morphological imaging. Histopathology preparations amounted to thirty-two slides from 4 groups, each consisting of 8 rats. Group 1 served as a control consisting of rats that were not exposed to cigarette smoke. Group 2, the conventional-cigarette smoke (CCS) group, was exposed to tobacco-cigarette smoke at three cigarettes/session with an estimated duration of exposure of 10 min and one day, carried out in 2 sessions (morning and evening); exposure was carried out for 30 days. Group 3, the electronic-cigarette smoke (ECS) exposure group, was exposed to electronic cigarettes with as much as 0.6 mL of liquid/session, with an estimated duration of 10 min and one day, carried out in 2 sessions; exposure was carried out for 30 days. Group 4 was subjected to transitional cigarette smoke exposure (TCS), with exposure to tobacco smoke, as per the regimen applied in the CCS group, for 15 days. They were then exposed to electronic-cigarette smoke, as per the regimen applied in the ECS group, for 15 days. In every session, the rats were exposed to cigarette smoke via a vacuum machine operated using a cycle of exposure for 5 s and a rest for 30 s until the tobacco cigarette ran out. Smoke exposure was set to 2 L/min with air supplied at 2 L/min, amounting to a total rate of 4 L/min [15].

Histopathological evaluation was performed both qualitatively, via microscopic description of morphological changes, and semi-quantitatively, using ImageJ (Version 1.54g, National Institutes of Health, Bethesda, MD, USA) software to measure the percentage of damaged tissue area. Microscopic observations were conducted under a light microscope at ×40 magnification to note detailed cellular changes and ×100 magnification to examine general tissue architecture. Images were captured using a digital camera attached to the microscope, and ImageJ was used for image acquisition and analysis to ensure reproducibility.

## 3. Results

### 3.1. HP Spleen Lesion Area

Table 1 shows the percentage of total histopathological damage obtained from ImageJ analysis across 10 microscopic fields of view.

According to the results of the the Kruskal–Wallis test, with respect to the necrosis parameter, the ECS group showed the highest mean rank, which was significantly higher than that of the control (*p* < 0.05), CCS (*p* < 0.05), and TCS (*p* < 0.05) groups, indicating the ECS group had the most severe degree of necrosis. For the degeneration parameter, the CCS group exhibited the highest mean rank, significantly higher than that of the control group (*p* < 0.05), followed by the ECS (*p* < 0.05) and TCS (*p* < 0.05) groups, indicating that degenerative changes were more pronounced in the exposed groups. In contrast, the hemosiderin, congestion, white pulp stretching, and vacuolization parameters showed relatively similar mean ranks across groups, with no statistically significant differences among the control, CCS, ECS, and TCS groups in any pairwise comparison (*p* > 0.05).

### 3.2. Histopathological Findings

**Figure 1:**
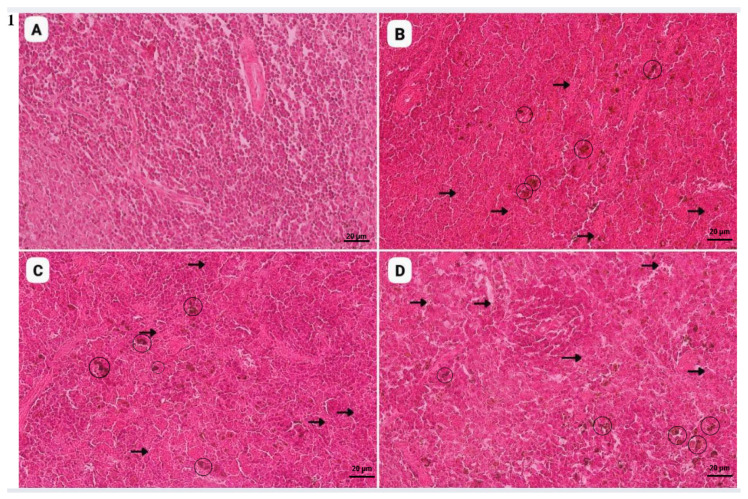

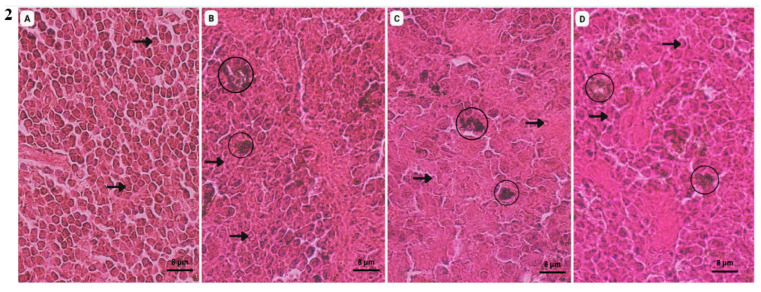
Hemosiderin (circle) and necrosis (arrow) were seen in the spleen in the treated groups. Dark-brown pigment formed from the breakdown of hemoglobin. The cell nucleus has undergone changes (compaction, rupture, and lysis). (Figure 1(1,2)) (**A**) Control; (**B**) conventional-cigarette smoke; (**C**) electronic-cigarette smoke; (**D**) transitional cigarette smoke. (Figure 1(1) H&E ×40, Figure 1(2) ×100).

**Figure 2:**
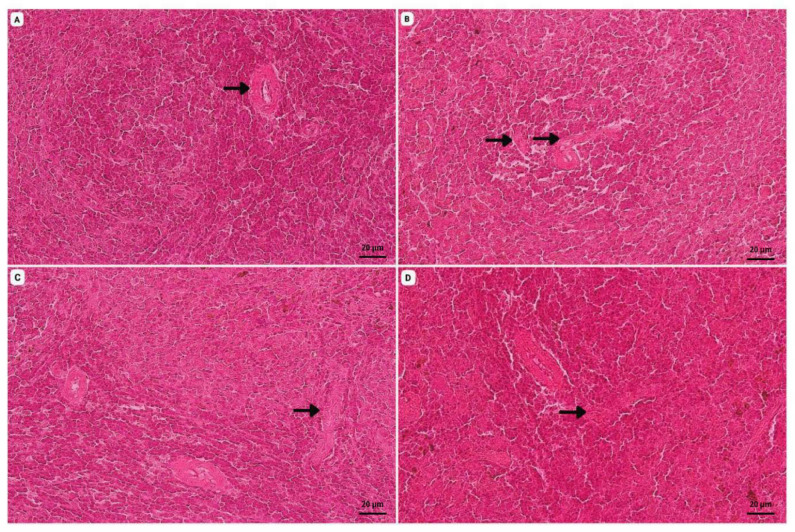
Blood vessel degeneration (arrow). Damage to the structure of blood vessels that are no longer intact. (**A**) Control; (**B**) conventional-cigarette smoke; (**C**) electronic-cigarette smoke; (**D**) transitional cigarette smoke (H&E, ×40).

**Figure 3:**
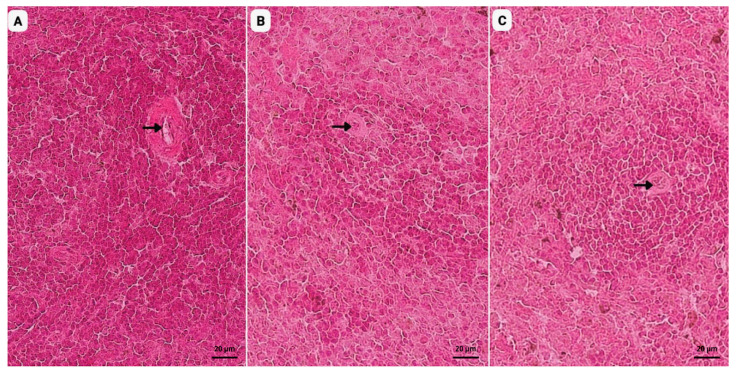
Congestion (arrow). Accumulation of blood within the blood vessels. (**A**) Control; (**B**) conventional-cigarette smoke; (**C**) electronic-cigarette smoke (H&E, ×40).

**Figure 4:**
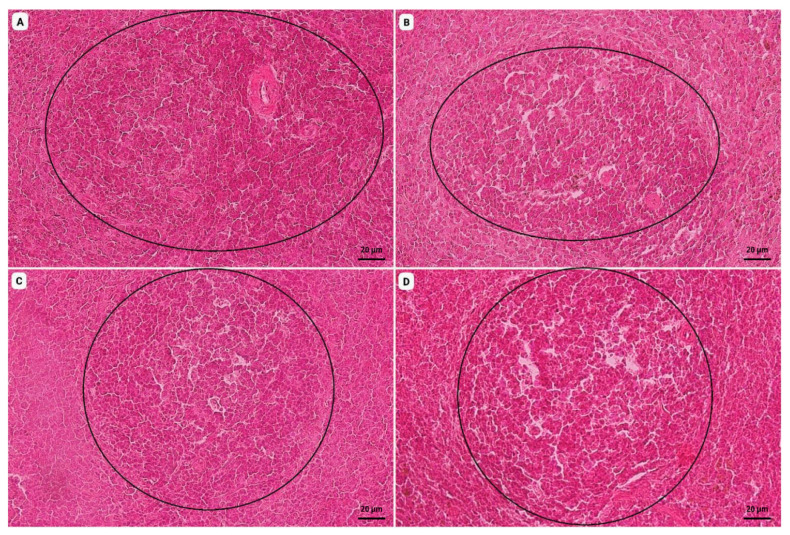
White-pulp dilatation (circle). White-pulp dilatation due to lymphoid depletion. (**A**) Control; (**B**) conventional-cigarette smoke; (**C**) electronic-cigarette smoke; (**D**) transitional cigarette smoke (H&E, ×40).

We obtained histopathological findings from 32 samples of spleen organs exposed to tobacco and electronic cigarettes and the transition between the two (Figure 1, Figure 2, Figure 3 and Figure 4).

## 4. Discussion

The entry of toxic substances from cigarette smoke into the body, which leads to spleen damage, begins with the combustion of cigarettes, producing smoke containing various harmful compounds. Tobacco-cigarette smoke contains nicotine, tar, carbon monoxide (CO), and other hazardous substances, whereas electronic cigarettes contain propylene glycol and vegetable glycerin, which, when heated, generate acrolein, formaldehyde, and heavy metal compounds. Acrolein and formaldehyde are highly reactive aldehydes that possess strong cytotoxic and immunotoxic properties, contributing to tissue injury in immune organs such as the spleen. Cigarette smoke enters the body through the respiratory tract, passing from the nasal cavity into the alveoli. The substances in cigarette smoke diffuse across the alveolar walls and enter the vascular system as free radicals, triggering oxidative stress that can damage cellular and tissue structure and function, ultimately resulting in organ damage [10]. These toxic substances subsequently circulate to various organs, including the spleen.

Within the bloodstream, toxic compounds from cigarette smoke come into direct contact with the vascular endothelium, thereby triggering inflammation and endothelial damage [17]. Free radicals present in cigarette smoke, including those from secondhand smoke (SHS), enter the blood vessels and reduce the production of nitric oxide (NO) by endothelial cells [18]. Nitric oxide is a key molecule involved in vasodilation; therefore, a decrease in NO levels due to free-radical exposure disrupts vasodilatory function and ultimately leads to endothelial dysfunction [17]. In addition, aldehydes such as acrolein and formaldehyde exacerbate endothelial injury by inducing oxidative stress, increasing vascular permeability, and promoting inflammatory signaling. Exposure to toxic compounds also induces mitochondrial depolarization in endothelial cells, which further exacerbates endothelial injury [19]. This endothelial damage results in the loss of vascular structural integrity and gradually promotes vascular degeneration, particularly when toxic exposure occurs continuously, including in the blood vessels of the spleen (Figure 2).

Nicotine entering the bloodstream also affects the central nervous system through the release of neurotransmitters, leading to vasoconstriction, increased blood pressure, tachycardia, and elevated cardiac output [20]. The resulting endothelial dysfunction stimulates the release of vasoconstrictors, causing narrowing of blood vessels and impaired blood flow, which ultimately leads to congestion [21]. Congestion represents a pathological response that is reversible when exposure to toxic substances is discontinued and vascular function is restored [22].

In addition, carbon monoxide present in cigarette smoke increases the formation of carboxyhemoglobin, thereby reducing the blood’s oxygen-carrying capacity and resulting in hypoxia [12]. The body responds to hypoxic conditions by increasing erythrocyte production as a compensatory mechanism [12,23]. This elevation in erythrocyte levels causes the spleen to work more intensively to break down aged or damaged red blood cells, leading to iron release and hemosiderin formation, ultimately resulting in hemosiderin accumulation in the spleen due to cigarette smoke exposure, a process that remains reversible [1,24]. In addition to hemosiderin accumulation, hypoxia also induces cellular necrosis, defined as cell death due to oxygen deprivation (Figure 1).

Necrosis may also be exacerbated by the effects of cigarette smoke, which reduces antioxidant levels in the body, thereby increasing oxidative stress and free-radical formation [25]. Free radicals induce lipid peroxidation of cell membranes, ultimately leading to cellular damage and necrosis (Figure 1). Acrolein and formaldehyde further intensify this process by depleting intracellular antioxidants and directly damaging cellular membranes, thereby accelerating lymphocyte loss in the splenic white pulp. Prolonged exposure to free radicals also promotes excessive cellular infiltration, resulting in damage to the lymphoid tissue of the spleen. Consequently, a reduction in white-pulp follicle density occurs due to pathological alterations in splenic cells, a process characterized by dilation of the white pulp as a result of decreased follicular density (Figure 4). These histological changes in the spleen reflect the activity and immune response of the body [26].

**Scheme 1:**
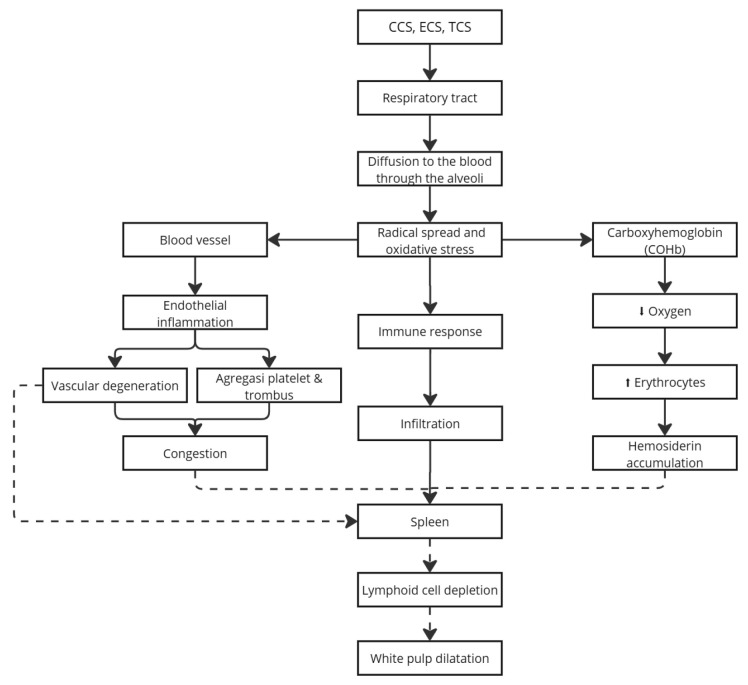
The effects of cigarette smoke on the spleen. ⬆ (Increase) and ⬇ (Decrease). CCS: conventional-cigarette smoke; ECS: electronic-cigarette smoke; TCS: transitional cigarette smoke. (American Lung Association, 2020 [35]; DiGiacomo et al., 2018 [17]; Wibowo et al., 2017 [12]; Ischorina et al., 2016 [23]; Estela et al., 2021 [36]; Marshall et al., 2018 [27]; Pisinger and Rasmussen, 2022 [28]; Nugrahaningsih et al., 2023 [26].

Based on the calculation of spleen damage severity, exposure to electronic-cigarette smoke (ECS) resulted in a lower degree of histopathological damage compared with conventional-cigarette smoke (CCS). However, the transitional cigarette smoke (TCS) group exhibited the highest level of damage, particularly in terms of the parameter white-pulp dilation. The damage observed in the TCS group was associated with impaired immunological function and structural alterations of the spleen, as reflected by lymphoid depletion and white-pulp dilation. This finding is critical because the spleen is a major lymphoid organ composed of B and T lymphocytes that play an essential role in adaptive immune responses, including antigen recognition and antibody production [27]. The pronounced damage in the TCS group may be explained by cumulative toxic effects resulting from sequential exposure to tobacco cigarette smoke followed by electronic-cigarette vapor. Transitional exposure increases the overall toxic burden through the combined presence of tobacco-specific nitrosamines (TSNAs), heavy metals, polycyclic aromatic hydrocarbons (PAHs), and volatile organic compounds (VOCs), as well as reactive aldehydes such as acrolein and formaldehyde, which are known to induce oxidative stress, endothelial injury, and immune dysregulation [4,28]. Acrolein, a reactive aldehyde found in conventional-cigarette smoke and electronic-cigarette vapor, impairs lymphocyte viability and suppresses cytokine production, contributing to immune dysfunction [29]. Acrolein also induces oxidative stress and inflammatory signaling, thereby enhancing immunotoxic effects in lymphoid tissues [30]. Formaldehyde exposure alters lymphocyte subpopulations and suppresses immune-related cytokine expression in the spleen while promoting oxidative stress and inflammation that may exacerbate splenic injury [31,32]. In addition, residual effects of carbon monoxide and other combustion-derived toxins from prior CCS exposure may persist during the ECS phase, limiting tissue recovery and instead amplifying oxidative and inflammatory damage [33]. Consistent with previous reports, dual or transitional use of tobacco cigarettes and electronic cigarettes exerts more severe immunotoxic effects than single-product exposure, indicating that switching from conventional cigarettes to electronic cigarettes does not necessarily mitigate, and may even aggravate, splenic injury [34] (Scheme 1).

## 5. Conclusions

Exposure to conventional-cigarette smoke, electronic-cigarette vapor, and their transitional use caused spleen damage in male rats, with the transitional exposure group exhibiting the most severe lymphoid depletion and marked enlargement of the white pulp. These findings indicate that switching from conventional tobacco cigarettes to electronic cigarettes does not mitigate spleen injury and may even exacerbate immunological impairment due to the combined effects of toxic exposure.

This study did not include a comprehensive chemical characterization of electronic-cigarette (ECS) vapor. Although the ECS device specifications and e-liquid composition were clearly described in the Methods Section, the composition of ECS aerosols is known to vary considerably depending on device type, e-liquid formulation, and operating conditions. Therefore, the present findings are specific to the ECS product and exposure conditions used in this study. Furthermore, this study evaluated only the transition from combustible-cigarette smoke (CCS) to ECS exposure. Other transitional sequences, such as ECS exposure followed by CCS, were not investigated and may produce different biological effects. Accordingly, these results should not be generalized beyond the specific exposure context examined.

## Figures and Tables

**Table 1:** Percentage of HP spleen organ lesion area.

Group	Control	CCS	ECS	TCS
A	1.10 ± 0.25	0.97 ± 0.12	1.09 ± 1.15	1.03 ± 0.15
B	0.31 ± 0.05	0.38 ± 0.04	0.55 ± 0.06	0.33 ± 0.26
C	0.02 ± 0.00	0.25 ± 0.13	0.07 ± 0.01	0.06 ± 0.01
D	0 ± 0.00	0.52 ± 0.52	0.02 ± 0.01	0 ± 0.00
E	1.14 ± 0.61	0.90 ± 0.55	1.28 ± 0.55	2.05 ± 1.67
F	0 ± 0.00	0.16 ± 0.14	0 ± 0.00	0 ± 0.00
Total	2.57	3.18	3.01	3.47

A: Hemosiderin; B: necrosis; C: vascular degeneration; D: congestion; E: dilatation of white pulp due to lymphoid depletion; F: vacuolization. Control; conventional-cigarette smoke (CCS); electronic-cigarette smoke (ECS); transitional cigarette smoke (TCS).

## Data Availability

The original contributions presented in this study are included in the article. Further inquiries can be directed to the corresponding author.

## Abbreviations

The following abbreviations are used in this manuscript:

CCS	Conventional-Cigarette Smoke
ECS	Electronic-Cigarette Smoke
TCS	Transitional Cigarette Smoke
CO	Carbon Monoxide
NO	Nitric Oxide
ROS	Reactive Oxygen Species
SHS	Secondhand Smoke
TSNA	Tobacco-Specific Nitrosamine
PAHs	Polycyclic Aromatic Hydrocarbons
VOCs	Volatile Organic Compounds
H&E	Hematoxylin and Eosin
PG	Propylene Glycol
VG	Vegetable Glycerin
HP	Histopathology
